# Clinical impact of transient lymphopenia

**DOI:** 10.1007/s10238-024-01340-0

**Published:** 2024-04-17

**Authors:** Luigi Petramala, Cinzia Milito, Francesca Sarlo, Adriana Servello, Francesco Circosta, Luca Marino, Germano Sardella, Piero Trapani, Giulio D’aguanno, Antonino Cimo’, Gioacchino Galardo, Claudio Letizia

**Affiliations:** 1 Department of Translational and Precision Medicine, “Sapienza” University of Rome, Rome, Italy; 2 Department of Molecular Medicine, “Sapienza” University of Rome, Rome, Italy; 3 UOC Chimica, Biochimica E Biologia Molecolare Clinica, Fondazione Policlinico Universitario A. Gemelli I.R.C.C.S, Rome, Italy; 4 Emergency Medicine Unit, Department of Emergency-Acceptance, Critical Areas and Trauma, Policlinico “Umberto I”, Rome, Italy; 5 Department of Clinical, Internal, Anesthesiological and Cardiovascular Sciences, “Sapienza” University of Rome, Rome, Italy; 6 Department of Mechanical and Aerospace Engineering, “Sapienza” University of Rome, Rome, Italy; 7 Department of Medico-Surgical Sciences and Biotechnologies, Sapienza University, Rome, Italy; 8 General Surgery Unit, ICOT Hospital, Latina, Italy

**Keywords:** Lymphopenia, Clinical frailty, Immunosuppression, Diabetes, COVID-19 infection

## Abstract

Transient or persistent immunosuppression is a known risk factor for morbidity and mortality in critically ill patients. Aim of the present study is to evaluate the lymphopenia in patients admitted to the Emergency Unit of AOU Policlinico Umberto I, to investigate its prevalence at admission and the persistence during hospitalization until discharge. Possible correlations were evaluated between lymphopenia, diagnosis of admission, comorbidities and chronic treatments. In this study, 240 patients (142 men; 98 female; mean age 75.1 ± 15.1) were enrolled. Patients were divided into two groups according to the lymphocytes count at hospital admission, namely “Group A” with lymphopenia and “Group B” with values in the normal range. Moreover, the patients in group A were distinguished in relation to the regression or persistence of the lymphopenia assessed at the time of hospital discharge (Group A1: persistence; Group A2: normalization). Prevalence of lymphopenia at admission was 57%; Group A showed higher mean age and percentage of patients over 65 years of age; and none differences were observed regarding gender. Prevalence of lymphopenia at admission was 57%; Group A showed higher mean age and percentage of patients over 65 years of age; no differences were observed regarding gender. All subsets of the lymphocytes (CD4^+^, CD8^+^, NK) were equally reduced. Persistent lymphopenia was found in 19% of patients. Lymphopenia should be valued at the time of hospital admission as a factor influencing the prognosis, the management and the treatment of these patients.

## Introduction

In the evaluation of the circulating cells that constitute the leukocytes, lymphocytes include T cells (about 75%), B cells (25%) and natural killer (NK) (about 5%). Lymphopenia or “lymphocytopenia” refers to a count of total lymphocytes < 1000/mcL (1 × 10^9^/L) in adults or < 3000/mcL (< 3 × 10^9^/L) in children < 2 years. Lymphopenia may be caused by primary conditions such as congenital immunodeficiency disorders, or by acquired causes including malnutrition, infectious diseases, sepsis, autoimmune and lymphoproliferative disorders, malignancies, medications (steroid, chemotherapy) and protein-losing conditions including severe burns, amyloidosis disease and inflammatory bowel disease.

In a large prospective cohort study in the general Danish population, it was investigated whether a low lymphocyte count could predict risk of later hospitalization or risk of death due to an infection. Even if physicians are generally not recommended to intervene in patients with lymphopenia without an associated diagnosed disease, lymphopenia in the general population is associated with a 1.4-fold increased risk of infection, increased risk of hospitalization due to acute infection such as pneumonia, skin infection, urinary tract infection, sepsis, diarrheal disease, endocarditis, cardiovascular disease and cancer and a 1.7-fold increased risk of infection-related death [1]. In all hospitalized patients including patients admitted in emergency care units, lymphocyte count is routinely measured although laboratory values and trends do not influence clinical management [2]. In critically ill patients, the immune response is a complex and dynamic process that can be altered due to various factors, and prolonged lymphopenia may be used as a marker of persistent immunosuppression.

In emergency and intensive care settings, persistent lymphopenia following the diagnosis of sepsis predicts early and late mortality and may be associated with worse prognosis in sepsis and community acquired pneumonia [3, 4]. In several studies conducted in the emergency care unit, prolonged lymphopenia was identified as a marker of persistent immunosuppression in septic patients showing that low absolute lymphocyte counts are predictive of postoperative sepsis and are a better predictor of bacteremia [5]. Moreover, in a study enrolling septic patients, although the absolute lymphocyte counts decrease to similarly low levels in survivors and non-survivors at the onset of sepsis, in non-survivors’ absolute lymphocyte counts remain persistently low while survivors experience lymphocyte recovery [4].

Thus, absolute lymphocyte count is a convenient biomarker for monitoring immune status but is also suitable for clinical application and for identifying critically ill patients at higher risk for poor prognosis. The dynamic monitoring of acute lymphocyte count is useful in patients admitted to the intensive care unit (ICU) and emergency unit [6]. It is a routine test helpful for grouping critically ill patients and for identifying patients at highest risk for immunosuppression and death.

On these bases, we aim to evaluate lymphocytopenia in patients admitted to the Emergency Unit of AOU Policlinico Umberto I. In particular, we investigated the prevalence of lymphocytopenia at admission, its persistence during hospitalization and eventually presence at discharge.

Moreover, we explored the possible correlation between lymphocytopenia and the diagnosis of admission, taking into account previous patients’ comorbidities, chronic treatments and other factors suggesting the frailty of patients (i.e., length of hospitalization, hospital morbidity and mortality, potential complications) and between lymphocytopenia and clinical and laboratory parameters (neutrophil-to-lymphocyte ratio (NLR), lymphocyte subsets and serum immunoglobulins levels).

## Materials and methods

### Study design

Between August 1, 2022, to July 2023, we consecutively enrolled 240 patients (142 men; 98 female; mean age 75.1 ± 15.1) admitted at the Emergency Department of AOU Policlinico Umberto I, for several acute illnesses. In overall patients, we have evaluated anthropometrics parameters, clinical data, and fasting venous blood samples. Complete blood cell count was performed for all patients on admission. Blood samples were collected between 05:00 and 07:00, and 4 ml of blood was drawn into EDTA tubes. The white blood cell counts, including lymphocyte counts, were measured on fresh samples shortly after the blood draw. This procedure was carried out at hospital admission and repeated at discharge. Lymphopenia was defined as an absolute lymphocyte count less than (1.0 × 10^^3^ cells/μL), which is the lower limit of normal at our institution. Patients were divided into two groups, “Group A” with lymphopenia and “Group B” with plasmatic lymphocytes in the normal range at hospital admission. Serum protein electrophoresis was performed on all patients; in those patients with hypogammaglobulinemia (gamma fraction on electrophoresis < 11%), the immunoglobulins counts (IgG, IgM, IgA) were requested. A lymphocyte flow cytometry analysis was also conducted, including the evaluation of CD4^+^, CD8^+^, CD16^+^/CD56^+^, and CD19^+^ subsets of lymphocytes in Group A patients. The duration of hospitalization, main comorbidities and therapy taken at the time of hospitalization were evaluated as clinical parameters. We evaluated a previous COVID-19 disease, reporting among the comorbidities an “previous COVID-19 disease” if reported in the previous 6 months.

Furthermore, the patients in group A were distinguished in relation to the regression or persistence of the lymphopenia assessed at the time of hospital discharge, distinguishing the patients in “Group A1” (persistence of lymphopenia) and “Group A2” (normalization of lymphocyte values).

Patients with active virus diseases (Human Immunodeficiency Virus, cytomegalovirus, Epstein-Barr virus and Infectious Mononucleosis), hematological malignancies (already known or newly diagnosed at hospital admittance) and acute COVID-19 disease were excluded from the study. Moreover, we excluded patients affected by primary conditions inducing lymphopenia (such as congenital immunodeficiency disorders, amyloidosis disease, and inflammatory bowel disease).

### Statistical analysis

All data are expressed as mean standard deviation (SD). Differences between means were assessed by the Student’s t test or the Mann–Whitney U test in non-normally distributed data for two-sample comparison, or by one-way analysis of variance (ANOVA) applying the Fisher least significant difference post hoc test for multiple comparisons. Chi^^2^ statistics were used to assess differences between categorical variables. Relationships between continuous variables were assessed calculating the Pearson correlation coefficient or the Spearman rank correlation coefficient when appropriate. *P* values < 0.05 were taken as statistically significant. Statistical analysis was performed using dedicated statistical software SPSS (Statistical Package for Social Sciences, software, version 24; SPSS Inc, Chicago, Illinois, USA) and GraphPad (version 5.0; GraphPad Software, Inc, La Jolla, California, USA).

## Results

**Fig. 1:**
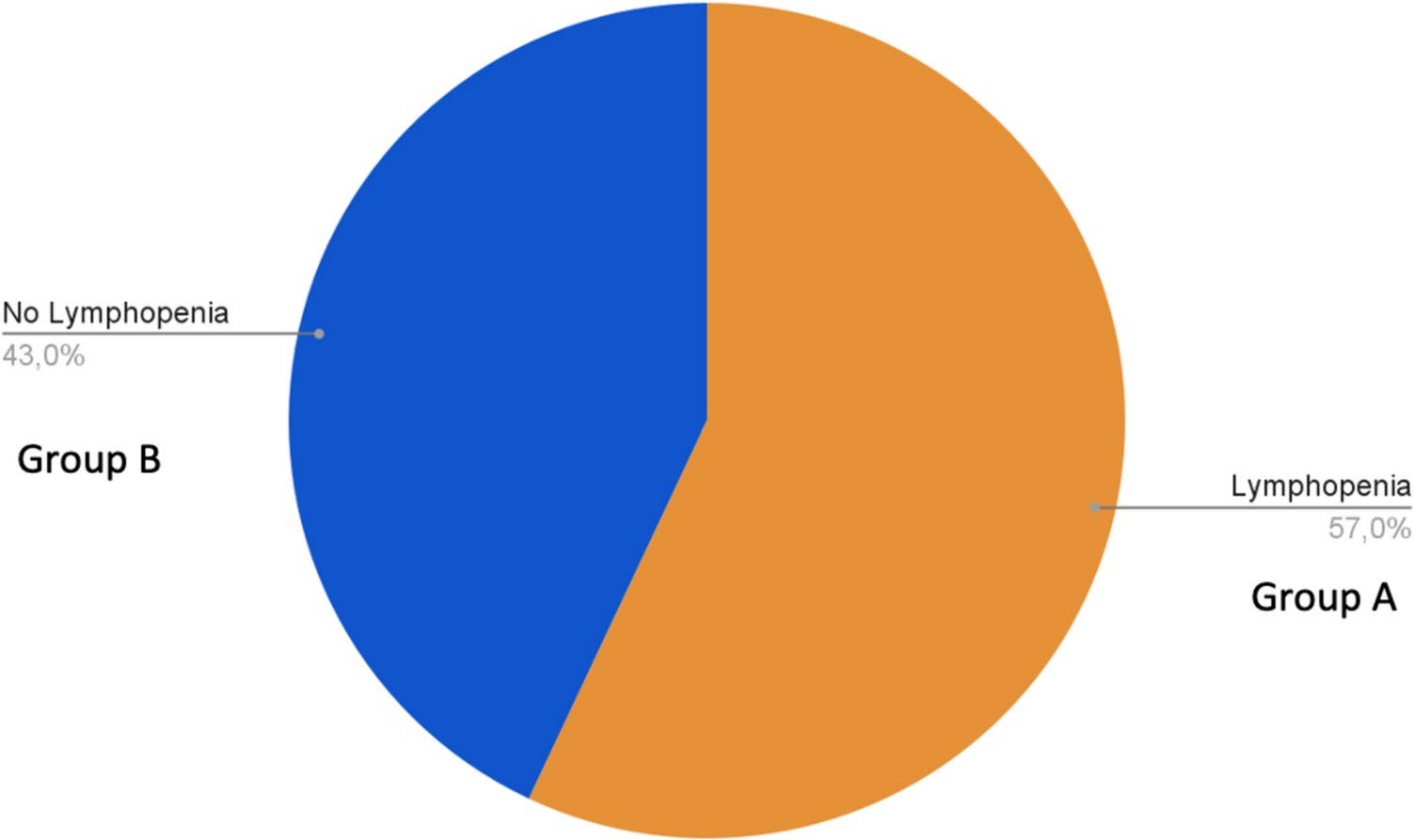
Prevalence of lymphopenia in patients enrolled at hospital admission. Group A: presence of lymphopenia. Group B: Normal lymphocyte count

A total of 240 patients (142 men; 98 females; mean age 75.1 ± 15.1) were enrolled consecutively in the study. They were divided into two groups: Group A (57%) exhibiting lymphopenia at admission in hospital and Group B (43%) without lymphopenia at admission (Fig. 1). In Table 1, we have reported clinical, anthropometric and biochemical characteristics of patients enrolled. In Group A we found significantly increased values of mean age (74.5 ± 14.6 years) and of the percentage of patients over 65 years of age (78%), compared to patients in group B (66.6 ± 15.6 years and 60%, respectively; *p* < 0.02); none differences were observed regarding gender.

**Table 1:** Anthropometric, biochemical and clinical data in patients enrolled

	Age (years)	Age > 65 years (%)	Gender (M %)	ANC (10^^3^ cells/μL)	ALC (10^^3^ cells/μL)	NLR (ratio)	Hypogammaglobulinemia (%)	Days of hospitalization (day)
Group A (n. 142)	74.5 ± 14.6	78	60.3	9.2 ± 7.1	0.74 ± 0.23	14.5 ± 1.3	7.8%	12.4 ± 8.6
Group B (n. 98)	66.6 ± 15.6	60	58.2	6.12 ± 3.8	1.8 ± 0.5	3.5 ± 2.2	10.9%	8.3 ± 5.1
*p* value	0.02	0.02	ns	0.001	0.001	0.001	ns	0.002

*ANC* absolute neutrophil count, *ALC* absolute lymphocyte count, *NLR* neutrophil-to-lymphocyte ratio, Hypogammaglobulinemia < 11%

Regarding neutrophils, group A showed a greater absolute number of circulating neutrophils (9.2 ± 7.1 10^^3^/μL, NLR (14.5 ± 1.3) compared to group B (6.12 ± 3.8 10^^3^/μL, 3.5 ± 2.2, respectively; *p* < 0.001). We did not find differences in gamma-globulin values. Regarding clinical data, patients of group A showed significantly longer duration of hospitalization in comparison with group B (12.4 ± 8.6 days vs. 8.3 ± 5.1 days; *p* < 0.002).

**Fig. 2:**
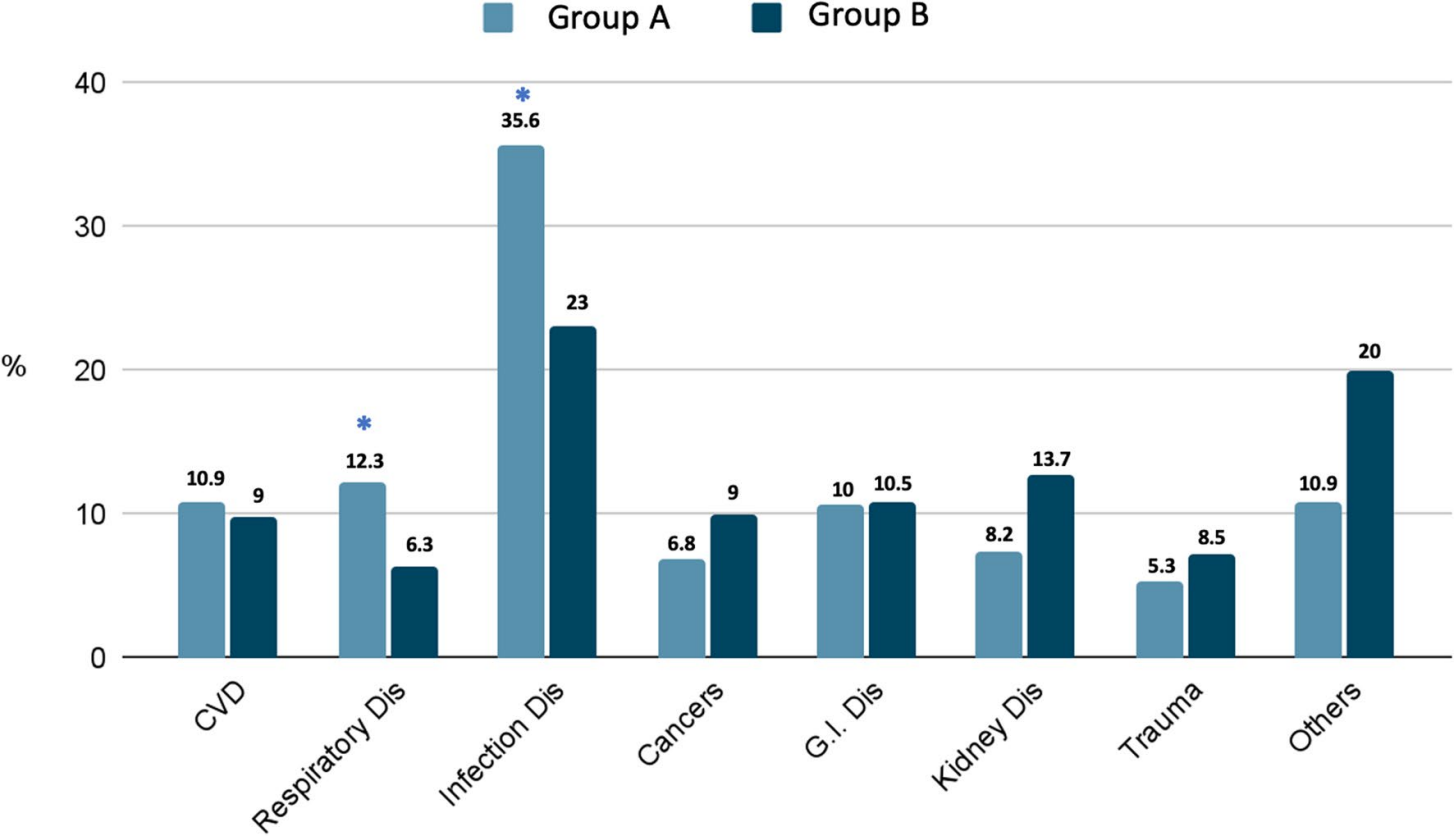
Diagnosis at hospital admission in patients enrolled. Group A: presence of lymphopenia. Group B: Normal lymphocyte count. *p < 0.05 versus Group B, G.I. Dis: gastro-intestinal diseases

Considering the diagnosis for hospitalization, in group A we have found significantly higher prevalence of respiratory diseases and infectious diseases (12.3% and 35.6%) compared to group B (6.3% and 23%, respectively; *p* < 0.05). No difference compared to other pathologies considered, such as CVD, cancers, pathologies of the gastrointestinal tract, kidney, trauma or other causes, was identified (Fig. 2).

**Fig. 3:**
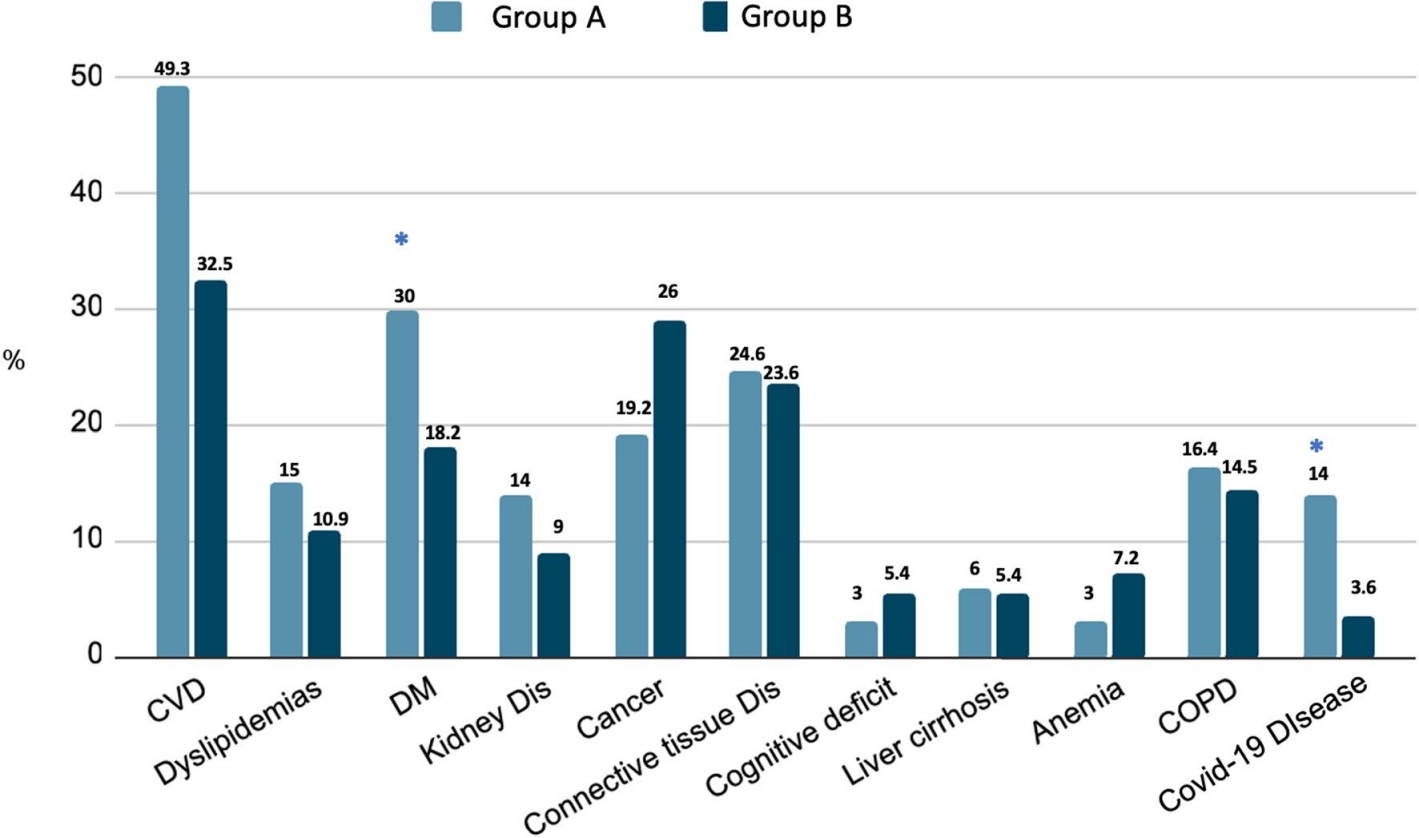
Comorbidities at hospital admission in patients enrolled. Group A: presence of lymphopenia. Group B: Normal lymphocyte count. *p < 0.05 versus Group B. *CVD* cardio-vascular diseases, *DM* diabetes mellitus, *COPD* chronic obstructive pulmonary disease

**Fig. 4:**
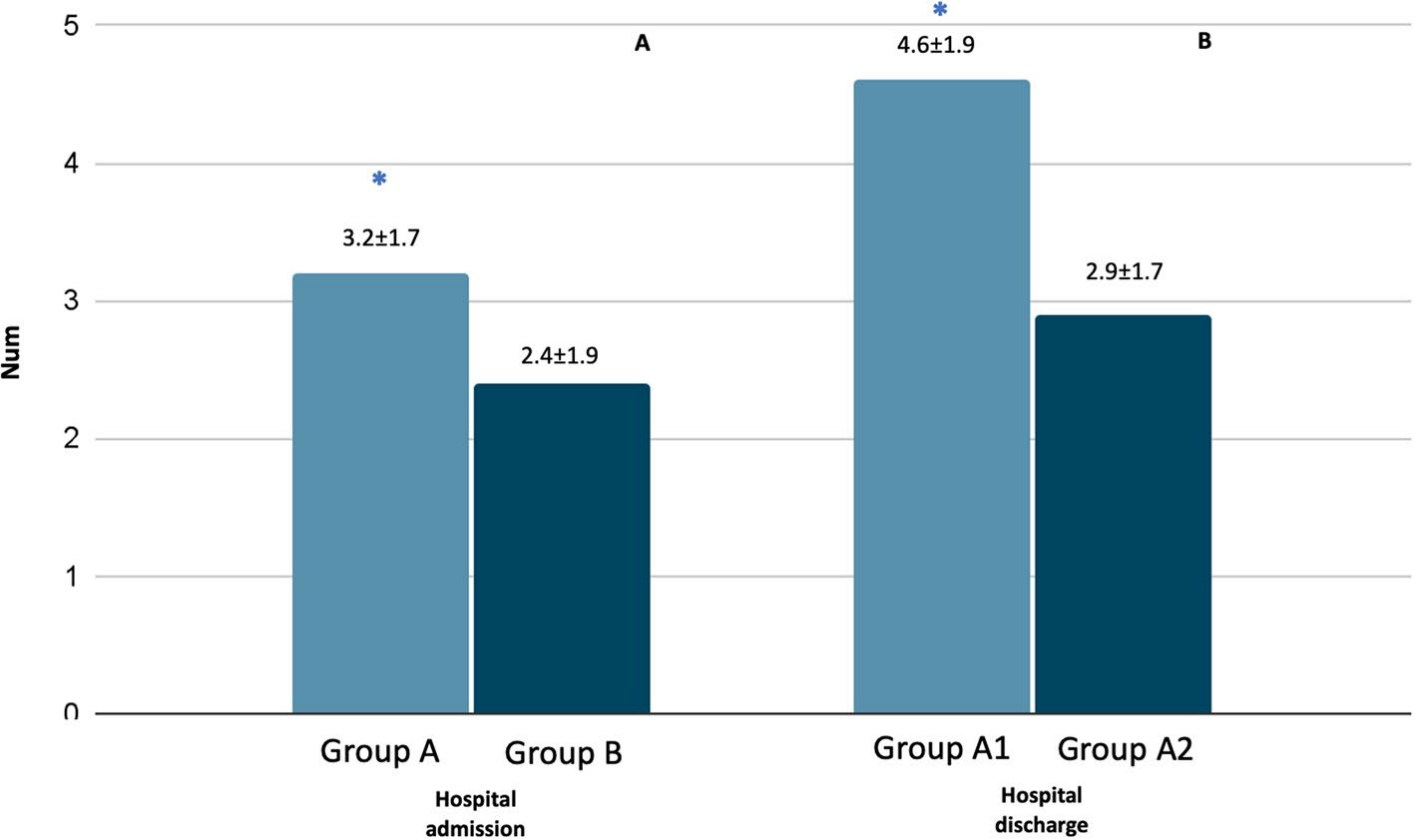
Average number of main comorbidities in patients enrolled at hospital admission (**A**) and at hospital discharge (**B**) in overall patients. Group A: presence of lymphopenia. Group B: Normal lymphocyte count. Group A1: Persistence of lymphopenia at hospital discharge. Group A2: Normalization of lymphocyte values at hospital discharge. *p < 0.05 versus Group B

Evaluating the comorbidities present at hospital admission, patients with lymphopenia showed higher frequency of diabetes mellitus (30%) and recent COVID-19 disease (14%) compared to patients without lymphopenia (18.2% and 3.6, respectively; *p* < 0.05) (Fig. 3). Moreover, in group A the mean number of comorbidities was significantly higher (3.2 ± 1.7) compared to group B (2.4 ± 1.9; *p* < 0.03) (Fig. 4a). No differences were observed regarding drug therapies possibly influencing immune response: Group A: steroids 6.8%, immunosuppressive medications 8.2%, chemotherapy 2.7% versus Group B 3.6%, 16.1% and 1%, respectively.

**Fig. 5:**
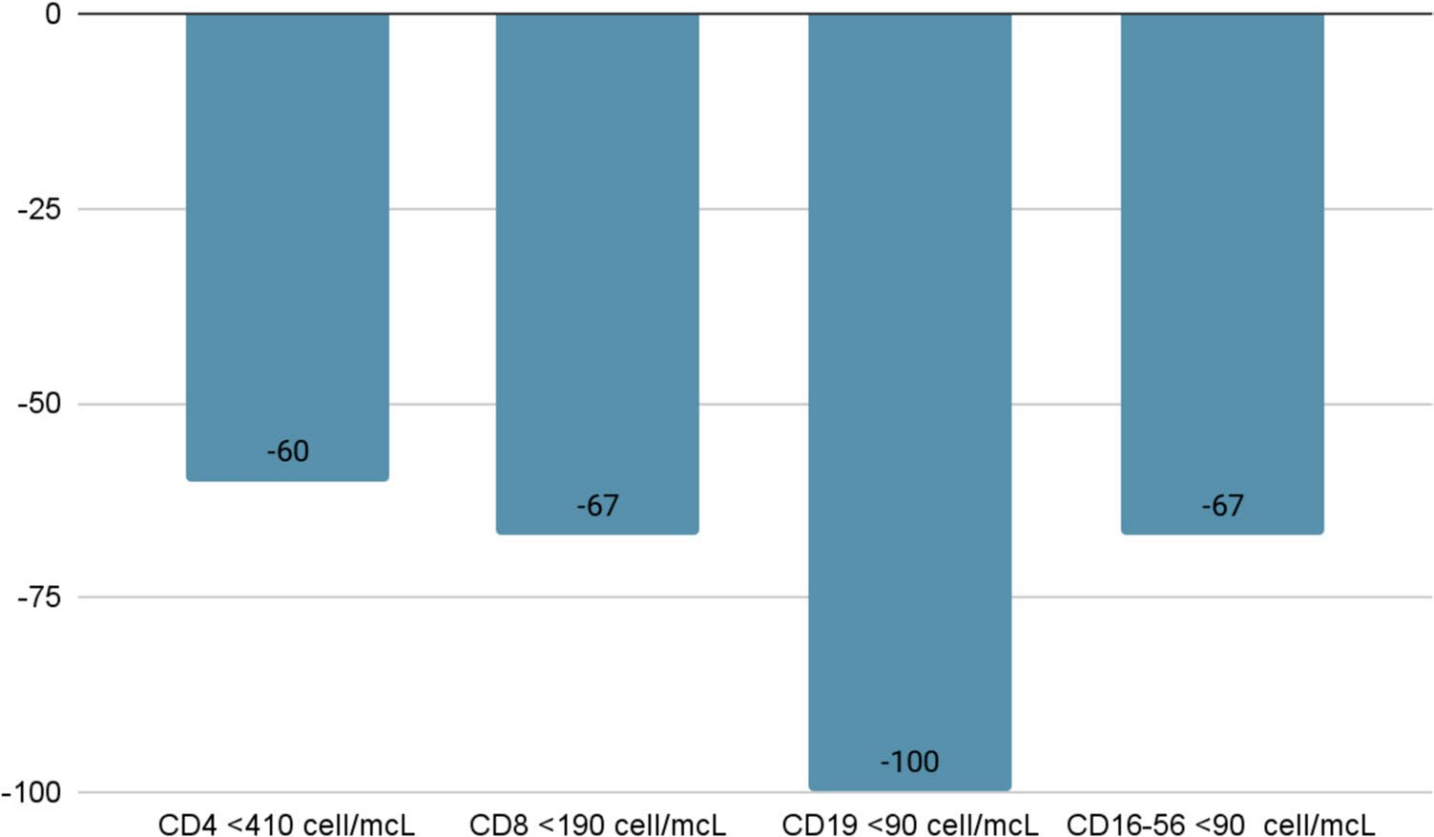
Prevalence of reduced values of different classes of lymphocytes through lymphocyte flow cytometry analysis in patients with lymphopenia (Group A) at hospital admission

In Group A, through lymphocyte flow cytometry analysis, we also evaluated the subset of the lymphocytes [B lymphocytes (CD19^+^), T lymphocytes (CD4^+^ and CD8^+^), and Natural Killer (CD16/56^+^)], highlighting that CD4^+^ T cells, CD8^+^ T cells and Natural Killer subgroups were equally reduced below the values of normality (60%, 67% and 67%, respectively), while CD19^+^ lymphocytes were reduced below normal values in all subjects (Fig. 5).

**Fig. 6:**
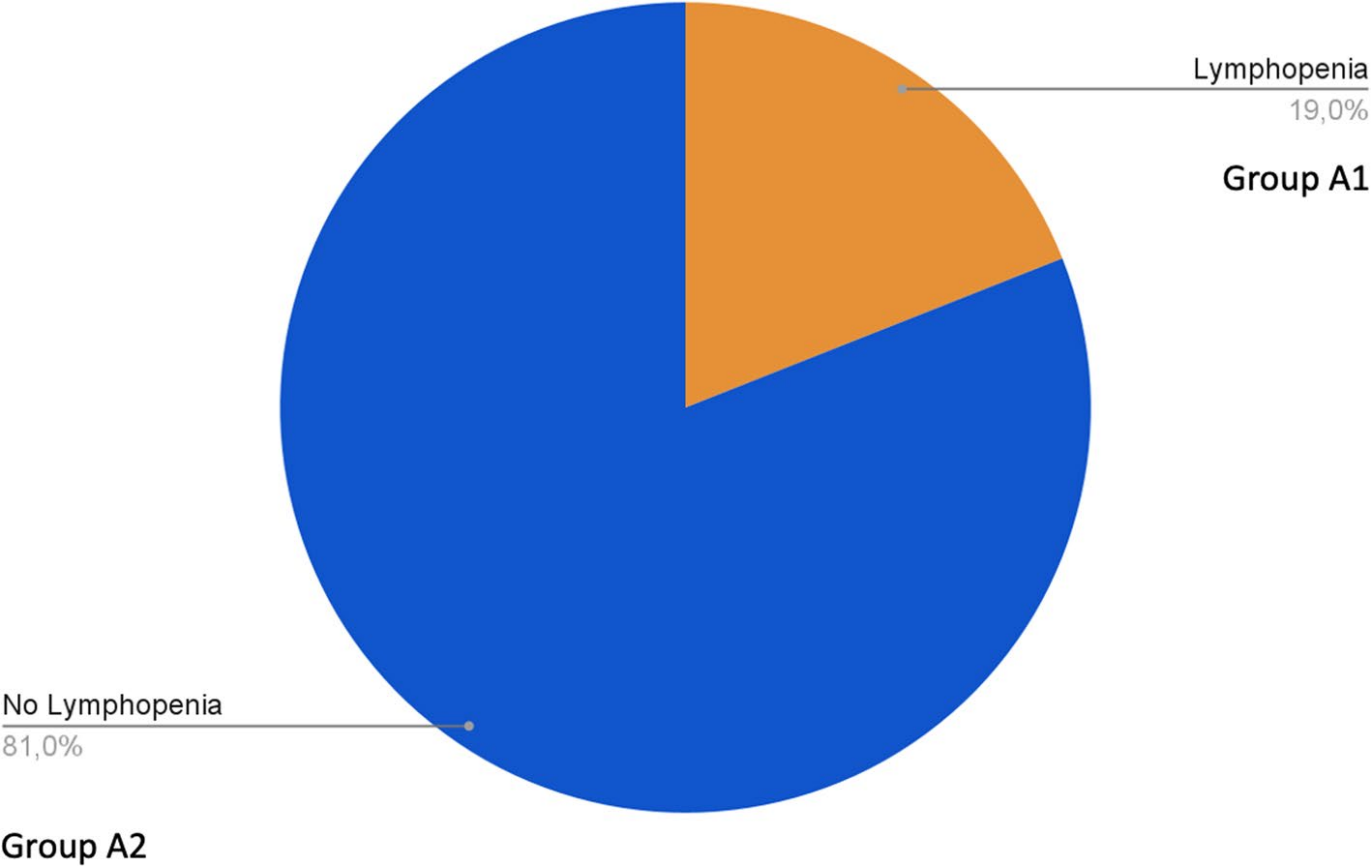
Prevalence of Lymphopenia at hospital discharge. Group A1: Persistence of lymphopenia at hospital discharge. Group A2: Normalization of lymphocyte values at hospital discharge

Re-evaluating patients with lymphopenia at admission, 19% of them showed persistent lymphopenia (Group A1), while in 81% we observed a normalization of lymphocyte values (Group A2) (Fig. 6).

**Fig. 7:**
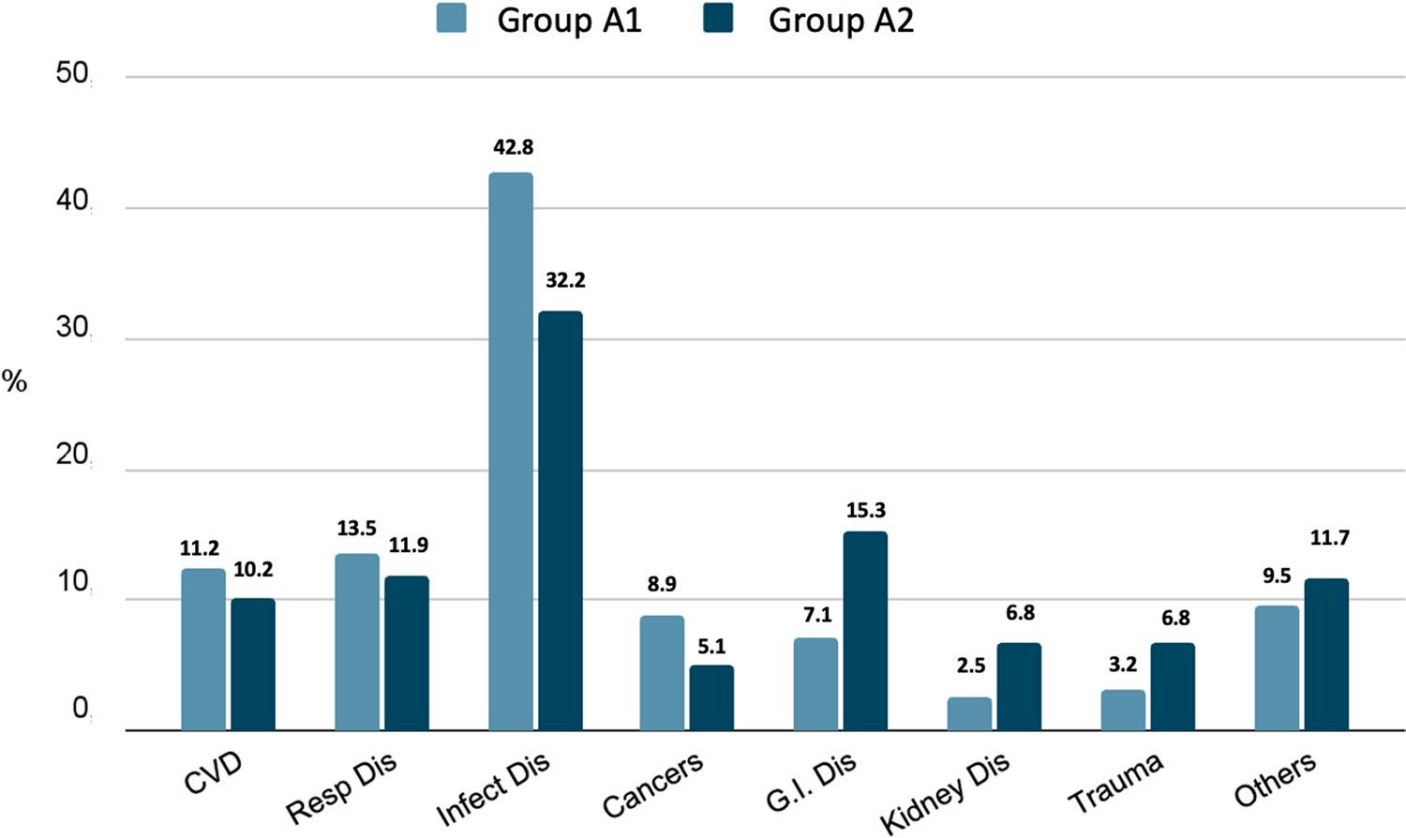
Diagnosis at hospital discharge in patients enrolled. Group A1: Persistence of lymphopenia at hospital discharge. Group A2: Normalization of lymphocyte values at hospital discharge. *CVD* cardio-vascular diseases, *DM* diabetes mellitus, *COPD* chronic obstructive pulmonary disease

**Fig. 8:**
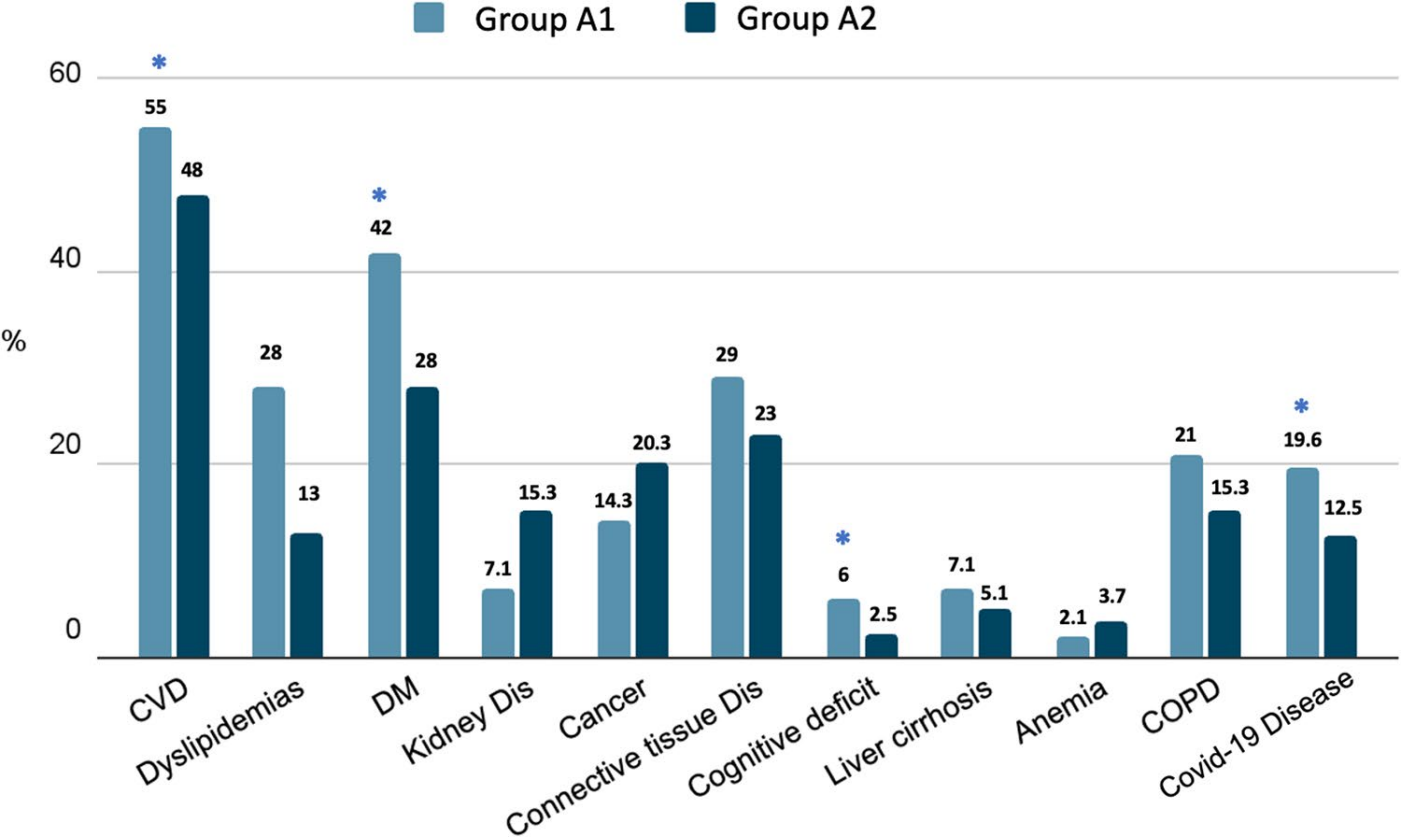
Comorbidities at hospital discharge in Group A patients in relation to persistence or remission of lymphopenia. Group A1: Persistence of lymphopenia at hospital discharge. Group A2: Normalization of lymphocyte values at hospital discharge. * *p* < 0.05 vs Group B. *CVD* cardio-vascular diseases, *DM* diabetes mellitus, *COPD* chronic obstructive pulmonary disease

In Groups A1 and A2 we did not highlight statistical differences regarding the pathologies causing hospitalization (Fig. 7); whereas, regarding the comorbidities, in group A1 we reported a greater prevalence of CVD (55%), diabetes (42%), cognitive defects (6%) and previous COVID-19 disease (19.6%) compared to group A2 (48%, 28%, 2.5% and 12.5%, respectively; *p* < 0.05) (Fig. 8). As regard, in group A1 the mean number of comorbidities was significantly higher (4.6 ± 1.94) compared to group B (2.9 ± 1.7; *p* < 0.05) (Fig. 4b). No differences were observed regarding drug therapy possibly having a negative influence on immunity response: Group A: steroids 7.1%, immunosuppressive medications 8%, chemotherapy 8%; Group B 6.8%, 10.2% and 3.4%, respectively.

## Discussion

Different studies show that transient or persistent immunosuppression is a risk factor for morbidity and mortality in critically ill patients [7]. Especially on subjects suffering from trauma, recent major surgery or sepsis, several authors shown that different parameters obtained from the blood count [i.e., absolute lymphocyte count (ALC) and lymphocyte subsets, such as CD4/CD8 and regulatory T cell] are suitable biomarkers for evaluating the immune function in critically ill patients and strongly correlated to prognosis [8, 9]. In clinical practice, ALC is one of the most briefly available biomarkers, reflecting the immune status in critically ill patients as well representing useful tool for screening patients in various immune regulation therapy conditions [10, 11]. It has long been established that lymphopenia increases the risk of infection and related death in hospitalized patients [1], especially in elderly patients or those admitted to Intensive Care Units (ICU) [12]. In particular, age represents an important risk factor, since the elderly are known to have blunted immunity, a condition responsible for specific organ failures, subverting the functions of immune cells [13].

In our series, conducted in an emergency unit, we reported a high prevalence of transient lymphopenia (more than half of patients), with significant correlations with clinical and biohumoral parameters.

According to persistence of lymphopenia, the trend of this parameter during hospital stay is also associated with different types of adverse clinical outcomes [14, 15]. As regard, in an extensive study, Pei Fei et al. have retrospectively evaluated over 10.000 critically ill patients admitted to ICU, highlighting that patients with persistent lymphopenia showed the highest incidence of negative outcomes [in-hospital mortality HR 1.44, 28-days mortality HR 1.66, development of catabolism syndrome HR 1.79, respectively) [6]. In the long-term prospective cohort study conducted in the Copenhagen General Population Study, Warny et al. found that patients with lymphopenia had higher mortality for all causes (HR 1.63), and, in particular, for CVD (HR 1.88), respiratory (HR 1.88) and infectious diseases (HR 1.86) [1].

In this regard, in our study, we highlighted that lymphopenia is more associated with a higher mean age, highly prevalent in patients > 65 years; moreover, lymphopenia has shown an important impact on some clinical aspects of the patient, such as length of hospital stay, identifying subjects with clinical fragility. Thus, ALC can be recognized as a useful marker easily evaluated through routine blood analysis at hospital admission and at discharge, becoming a worthwhile tool useful to personalized clinical management, to better stratify the risk of patients’ fragility, to target treatment up to adjuvant therapies potentially stimulating lymphocytes.

Infectious diseases, up to sepsis, represent a significant phenomenon related to the host–pathogen interaction, in which the immune response has an important role in determining critically ill patients. In particular, it often leads to identifying major defects in immunity during recovery, conferring increased susceptibility to secondary infections and leading to worsened outcomes [16, 17]. The intensity of the inflammatory response is mainly determined by the patient's background, such as comorbidities (e.g., cancer and hematological malignancies, solid organ transplant, autoimmune and systemic diseases, HIV, renal insufficiency, and liver failure, chronic alcoholism, malnourishment), use of immunosuppressive drugs, as well as the acute event that induces hospitalization [18].

In our study, we demonstrated that subjects with transient lymphopenia were more likely to have infectious and respiratory diseases as diagnosis for hospitalization.

Infection diseases can induce multiple defects both in innate and adaptive immunity including apoptosis-induced depletion of immune effector cells (lymphocytes and dendritic cells), monocyte deactivation, T cell exhaustion, increased myeloid-derived suppressor cells, and increased T regulatory cells [7].

In large population, Juan Carlos Andreu-Ballester et al. have found an high prevalence of lymphopenia (41%) during hospital stay, especially in patients with infectious diseases, highlighting that lymphopenia was closely correlated with higher in-hospital and post-discharge mortality; evaluating the relationship of lymphopenia with the four levels of the severity of illness and the risk of mortality, these authors found that lymphopenia was related to worse indexes at the time of hospital admission [19].

Community-acquired pneumonia (CAP) is an important infectious disease causing sepsis, characterized by high in-hospital mortality (4–14%) and significant development of multi-organ failure [20]. In CAP, the host response has been mainly focused on innate immunity and the inflammatory response [21, 22]. Recently, CAP associated with lymphopenia (L-CAP) has been identified as an independent risk factor for 30-day mortality [23]. As regards, in a large population of patients hospitalized for CAP, Mendez et al. found lymphopenia in over 39% of patients, characterized by decreased levels of all lymphocyte subsets, with partial recovery of CD4^+^ and CD8^+^ cells at day 4. Moreover, L-CAP patients presented a worse severity of systemic inflammation (higher levels of proinflammatory, granulocyte colony-stimulating factor, and monocyte chemoattractant protein-1) [22].

In our study, we identified the persistence of lymphopenia in a significant percentage (19%) of patients at hospital discharge, especially in subgroups with a significantly increased mean number of comorbidities, such as diabetes mellitus, previous COVID-19 infection, CVD and cognitive deficit.

Physiologically, infection diseases are associated with an immunological response with consequent activation and increase of T lymphocytes (both CD4^+^ and CD8^+^), B lymphocytes and natural killer cells, although after viral infections lymphocyte counts can be reduced [24]. As regards, possible causes can be related to consumption of lymphocytes, direct viral damage to lymphocytes, apoptosis of lymphocytes and immunosuppressive effects of the virus [25].

It has been well described that acute COVID-19 disease, in up to 50% of patients, is associated with a reduction in lymphocyte count, in particular the absolute counts of T lymphocytes (CD4^+^ and CD8^+^), suggesting that T cell immune function of COVID-19 patients is weakened [26] and associated with poor outcomes [27].

Recent studies have focused attention on the relationship between acute COVID-19 disease and diabetes mellitus (DM); as regard, Wu et al. have evaluated the circulating levels of lymphocytes in patients hospitalized for acute COVID-9 disease, highlighting that the diabetic subgroup showed a reduction in the average levels of circulating lymphocytes (50%), earlier onset of lymphopenia (52%) and greater duration of hospitalization (20%), compared to non-diabetic patients [28]. Several studies showed that DM patients often may present immune impairment, particularly concerning reduced levels of T lymphocyte (both CD4^+^/CD8^+^) and Natural Killer lymphocytes, suggesting decreased host defense to infectious diseases [29, 30]. Furthermore, the acute COVID-19 disease uses angiotensin converting enzyme 2 (ACE2), expressed by epithelial cells of the lung, intestine, and kidney for cellular internalization; in DM patients, increased expression of ACE2 enzyme has been shown, suggesting a greater susceptibility [31], as well as a consensual reduced activity of the T lymphocytes [32].

While acute effects of acute COVID-19 on the immune system have been studied, long-term impacts of SarS-CoV-2 on the cellular immune system remain to be analyzed. In an interesting article, Liu et al. have evaluated the immunological characteristics of peripheral blood mononuclear cells in convalescent patients after 2 months from acute COVID-19 disease, highlighting several morphological and functional aspects, including significant decreases in frequencies of invariant NKT and NKT-like cells, increased expression of Ki67 and TIM-3 on both CD4^+^ and CD8^+^ T cells, and reduced cytotoxic potential of T cells and NKT-like [33].

In our cohort, among the main comorbidities already present at hospital admission and associated with the persistence of lymphopenia, CVDs showed a significant prevalence.

Muthiah Vaduganathan et al. have performed a post hoc analysis of the EVEREST trial, conducted on hospitalized patients with worsening heart failure (HF) and ejection fraction (EF) ≤ 40%, evaluated during a 1-year follow-up, focusing attention on relationship between lymphocyte count with post-discharge outcomes. These authors found that patients with lymphopenia were older and with higher rates of comorbidities (diabetes mellitus, atrial fibrillation, and kidney insufficiency) and were clinically characterized by wide QRS duration, high natriuretic peptides, and low EF. Although lymphopenia during hospitalization was normalized in the majority of patients in the early post-discharge period, interestingly mild lymphopenia was associated with an increased all-cause mortality (HR 1.31), cardiovascular mortality or HF hospitalization at 3 months from hospital discharge (HR 1.14) [34].

Beyond hospitalized patients, circulating levels of lymphocytes in outpatients with chronic HF predict survival up to 1 year [35]. In last decades, several possible mechanisms have been proposed explaining the relationship between lymphopenia and CVD and HF; among these the main suggested elements were hemodynamic features, such as elevated bi-ventricular filling pressures, splanchnic congestion, with direct enteric losses of lymphocytes or leukocyte redistribution [36]; immunological features, such as strong immune activation, release of cytokines (i.e., tumor-necrosis factor-1), and apoptotic mechanisms, directly inducing reductions in lymphocyte counts (particularly T-helper cell and B-cell) (27–28); hormonal features, such as the activation of the hypothalamic–pituitary–adrenal axis inducing increased endogenous production of cortisol and catecholamines [34].

In summary, lymphopenia should be valued at the time of hospital admission by physicians as a factor influencing the prognosis, the management and the treatment of these patients. It is useful to pay high attention, especially to subjects with persistent lymphopenia during hospitalization, in order to identify subgroups of patients at higher frailty who require a closer monitoring to avoid a bad prognosis.
